# HDAC/IKK inhibition therapies in solid tumors

**DOI:** 10.18632/oncotarget.17512

**Published:** 2017-04-28

**Authors:** Ivana Vancurova, Himavanth R. Gatla, Ales Vancura

**Affiliations:** Department of Biological Sciences, St. John's University, NY, USA

**Keywords:** HDAC, IκB kinase, IL-8/CXCL8, ovarian cancer, solid tumors

**Figure 1:**
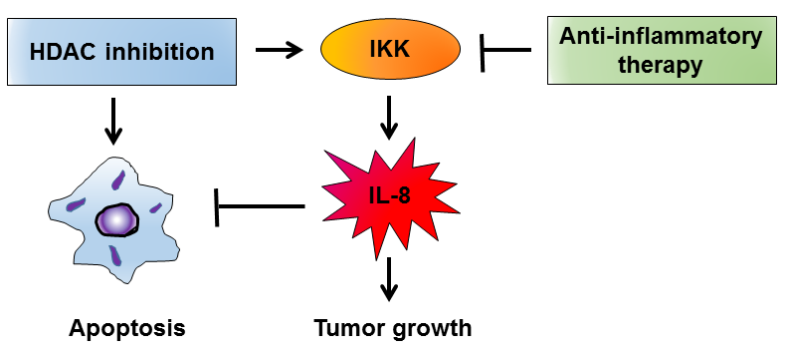
Combination of HDAC and IKK inhibitors decreases IL-8 expression in EOC cells, thus suppressing their survival and tumor growth. In EOC cells, HDAC inhibition induces IKK-dependent expression of IL-8, which increases EOC cell survival and proliferation. Inhibition of IKK activity suppresses the IL-8 expression, thus potentiating the effectiveness of HDAC inhibitors in reducing tumor growth.

Increased expression of the pro-angiogenic chemokine interleukin-8 (CXCL8, IL-8) contributes to the progression of solid cancers through its induction of tumor cell survival, proliferation, and metastasis. While histone deacetylase (HDAC) inhibitors have been remarkably effective in treating hematological malignancies, they have been far less effective as single agents in the treatment of solid tumors. We have recently reported results that may explain the limited efficiency of HDAC inhibitors in epithelial ovarian cancer (EOC), based on our data demonstrating that HDAC inhibition induces expression of IL-8 in EOC cells, resulting in their increased viability and proliferation, and that this is dependent on IκB kinase (IKK) activity [1]. Importantly, our *in vivo* results have demonstrated that combining HDAC and IKK inhibitors significantly reduces ovarian tumor growth when compared to either drug alone [1]. These data provide the first *in vivo* evidence demonstrating that IKK inhibition increases effectiveness of HDAC inhibitors in suppressing solid tumor growth, and suggesting that using IKK inhibitors may increase effectiveness of HDAC inhibitors in treating ovarian cancer and other solid tumors characterized by increased IL-8 expression (Figure 1).

Even though HDAC expression is increased in many types of solid tumors, including EOC, clinical trials targeting the HDAC activity in solid tumors have been disappointing [2, 3]. The rationale for developing HDAC inhibitors as anti-cancer agents was based on their ability to induce hyperacetylation of histones and non-histone proteins, resulting in increased differentiation, apoptosis, and cell cycle arrest of cancer cells [3, 4]. Vorinostat (suberoylanilide hydroxamic acid, Zolinza) and romidepsin (depsipeptide, FK228, Istodax) are the first HDACs inhibitors that have been approved by the FDA for the treatment of cutaneous T cell lymphoma. However, accumulating evidence indicates that HDAC inhibitors also activate the NFκB pro-survival pathway, which limits their lethality and contributes to drug resistance [5].

Interestingly, we have found that among the tested NFκB-regulated genes, only IL-8 is significantly induced by HDAC inhibition in EOC cells [1]. The induced IL-8 expression is associated with a gene specific, IKK-dependent p65 NFκB recruitment to the IL-8 promoter. However, why HDAC inhibition induces p65 recruitment only to the IL-8 promoter in EOC cells? One possible scenario is that in EOC cells, the IL-8 promoter is occupied by an HDAC, which prevents or limits the IL-8 transcription. Inhibition of HDAC activity would then facilitate histone acetylation, followed by p65 recruitment and induction of IL-8 transcription. Studies are currently in progress to identify the HDAC that specifically silences the IL-8 transcription in EOC cells. Since suppression or neutralization of the IL-8 induced by HDAC inhibitors increases their pro-apoptotic and anti-proliferative effect in EOC cells [1], these results indicate that the induced IL-8 expression may represent one of the mechanisms responsible for the limited effectiveness of HDAC inhibitors in ovarian cancer. This is supported by previous studies demonstrating that suppression of IL-8 reduces tumor growth of EOC cells [6, 7].

Targeting IKK activity and NFκB-dependent transcription of pro-survival genes induced by HDAC inhibition has been investigated in the treatment of multiple myeloma and other hematological malignancies [5]. In ovarian cancer and other solid tumors, combination of IKK and HDAC inhibitors has never been considered, perhaps because of their limited effectiveness as single agents. Our data indicate that by suppressing the IL-8 expression, IKK inhibitors may increase effectiveness of HDAC inhibitors in EOC. Future studies and clinical trials should examine the effect of IKK inhibitors on increasing the effectiveness of HDAC inhibitors in EOC and other solid cancers characterized by the increased IL-8 expression.
